# Application-Based Production and Testing of a Core–Sheath Fiber Strain Sensor for Wearable Electronics: Feasibility Study of Using the Sensors in Measuring Tri-Axial Trunk Motion Angles

**DOI:** 10.3390/s19194288

**Published:** 2019-10-03

**Authors:** Ahmad Rezaei, Tyler J. Cuthbert, Mohsen Gholami, Carlo Menon

**Affiliations:** Menrva Research Group, Schools of Mechatronic Systems & Engineering Science, Simon Fraser University, Metro Vancouver, BC V5A1S6, Canada; ahmad_rezaei@sfu.ca (A.R.); mohsen_gholami@sfu.ca (M.G.)

**Keywords:** fiber, strain sensor, core–sheath, piezoresistive, resistive sensor, random wave pattern, machine learning, trunk motion tracking, wearable sensors, smart shirt, random forest regressor

## Abstract

Wearable electronics are recognized as a vital tool for gathering in situ kinematic information of human body movements. In this paper, we describe the production of a core–sheath fiber strain sensor from readily available materials in a one-step dip-coating process, and demonstrate the development of a smart sleeveless shirt for measuring the kinematic angles of the trunk relative to the pelvis in complicated three-dimensional movements. The sensor’s piezoresistive properties and characteristics were studied with respect to the type of core material used. Sensor performance was optimized by straining above the intended working region to increase the consistency and accuracy of the piezoresistive sensor. The accuracy of the sensor when tracking random movements was tested using a rigorous 4-h random wave pattern to mimic what would be required for satisfactory use in prototype devices. By processing the raw signal with a machine learning algorithm, we were able to track a strain of random wave patterns to a normalized root mean square error of 1.6%, highlighting the consistency and reproducible behavior of the relatively simple sensor. Then, we evaluated the performance of these sensors in a prototype motion capture shirt, in a study with 12 participants performing a set of eight different types of uniaxial and multiaxial movements. A machine learning random forest regressor model estimated the trunk flexion, lateral bending, and rotation angles with errors of 4.26°, 3.53°, and 3.44° respectively. These results demonstrate the feasibility of using smart textiles for capturing complicated movements and a solution for the real-time monitoring of daily activities.

## 1. Introduction

Wearable electronics have become increasingly popular with the advancement of materials and electronics over the past two decades, producing more flexible and integrated functional materials. Wearable electronics span a wide variety of applications such as biomedical monitoring (i.e., body vitals), motion tracking, and integrated personal electronics [1]. Electronic components have become increasingly smaller, and have been designed for incorporation into wearable devices in fiber morphologies, including applications in energy harvesters [2,3,4,5,6], energy storage [2,7,8,9,10], and sensors [1,11]. Fiber sensors have been realized for strain [12,13,14], pressure [15,16,17], and chemical monitoring [18]. While there are non-wearable technologies that are able to track movement accurately (i.e., electromagnetic tracking and optoelectronic motion capture [19]), they have limitations that restrict their application in the workplace environment (sophisticated hardware, high cost, non-portable, limited capture space). Flexible fiber strain sensors have the potential for seamless integration into textiles and clothing creating wearable systems for in situ tracking of movements and further quantitative physical exposure measurement for injury risk assessment, with increased mobility and comfort, and without spatial restrictions—such as those with motion capture systems.

Besides flexible fiber sensors, inertial measurement units (IMU) have been a popular wearable alternative to alleviate the limitations of non-wearable systems. IMU sensors are microelectromechanical sensors consisting of an accelerometer, gyroscope, and magnetometer, which are able to capture the orientation of the sensor with respect to the earth-fixed reference coordinate system [20]. Several IMU units can be used together to form a wearable inertial motion capture (IMC) system. Recently, IMC systems have been used in several kinematic information measurement applications, tracking the lower extremities [21,22], spine [23,24], and trunk [25]. However, important performance limitations of IMCs have been reported [26,27]. The IMC performance accuracy was shown to decrease significantly by the movement characteristics (movement complexity, range, speed, and period) [20,26,27,28,29]. Fast complex movements over long periods of time have higher errors compared to short simple tasks [27,30]. In addition, magnetometers are extremely sensitive to environmental ferromagnetic disturbances and cause a considerable amount of error in IMU heading estimation [31,32]. These limitations have hindered the more widespread use of IMC systems in three-dimensional (3D) motion tracking, and therefore, the application of smart textiles employing flexible fiber strain sensors has been investigated as a substitute solution.

Flexible fiber strain sensors are commonly built on conductive components (i.e., metals [13,33], graphene [34,35], carbon nanotubes [36], carbon black [37], polymers [38,39], and ionic liquids [40,41]) and flexible polymers or substrates, as either composites or layered structures. There are a number of current methods to create layered coaxial fibers—with up to four layers—including melt spinning/extrusion [42], wet spinning [43,44], electrospinning [45,46], and newer technologies such as pressurized gyration spinning [47,48], solution blowing [49], and the use of 3D printers [50]. The advancement of manufacturing precise multilayered fibers will inevitably lead to more advanced multifunctional materials.

Thus far, fiber sensors have been produced using either resistive or capacitive sensing mechanisms [1,11,51]. Resistive sensors are attractive because of their low cost, low power consumption, ease of production, and non-specialized signal processing [52]. Piezoresistive sensors that utilize a polymer composite rely on the disconnection–reconnection of conductive pathways within the material when strain is applied [52]. This destructive mechanism often results in signal drift (i.e., an increase in the baseline resistance) and changing gauge factors, since conductive pathway reconnection may not occur. There have been a few recent examples using rubber elastic support core materials with piezoresistive sheaths to create high-performance fiber sensors. The piezoresistive sheaths have been comprised of carbon nanotube forests [36,53], embedded graphene nanoplatelets [54], silver nanowires/poly(vinylidenefluoride-co-trifluoroethylene) [55], carbon nanotube–silicone rubber composites [44], and elastomer-wrapped carbon nanotube fibers [56]. However, the effect of the core material properties on sensor performance has yet to be studied for piezoresistive thermoplastic elastomer polymer composites.

Thus far, accurately tracking three-dimensional (3D) kinematic motion beyond proof-of-concept testing using fiber strain sensors has been limited. Reports of kinematic tracking using piezoresistive sensors have targeted the wrist, hand, trunk, and knee gait [37,57,58], as well as a full body system. The potential of smart sensing textiles for angle measurement in planar movements and joints with one degree-of-freedom such as knee and fingers has been well established. However, the feasibility of these systems for angle measurement in complex multiplanar movements has yet to be evaluated. Bridging the gap between new sensor production and larger cohort studies researching accuracy and applicability is required to allow this field to flourish. Indeed, testing sensors using strains and strain rates that mimic what prototype devices would be ultimately tracking allows screening and optimization prior to creating these devices to ensure that sensor performance will be satisfactory. Sensor performance metrics beyond basic characterization are also important. For instance, testing scenarios that indicate how well a sensor will perform when exposed to real-world random movements is beneficial so that the applied research can focus on other factors such as sensor placement, the number of sensors required, and the signal processing to obtain the optimal and the highest quality kinematic motion information possible.

Herein, we outline the fabrication, characterization, and application-based optimization of core–sheath strain sensors with readily available starting materials that are easy to produce, and amenable to current fiber/textile production methods; then, we utilize this sensor to develop a smart shirt for accurately measuring the three-dimensional kinematic angles of the trunk.

The sensor was fabricated using two cores (a thermoplastic elastomer and an elastic core) that were hypothesized to support the sensing piezoresistive sheath and negate the negative effects of the addition of the conductive additives (i.e., lower elasticity, higher rigidity). In this case, we chose to target the motion tracking of the lower back angles that would be associated with a prototype device, allowing the optimization of the sensor for the strains associated with this location of the body. This type of specific application was intended to highlight that although many reports of new fabrication methodologies for fiber-based sensors are being reported, quite often, the actual performance of those sensors in a tracking application may not be sufficiently accurate. We used a two-prong approach. First, we determined the limits of the materials we were using with respect to their mechanical and piezoresistive properties. Second, we approached the problem from the requirements of the intended application, and tested the material within this working range to mimic use in a prototype device and determine if the sensor would perform sufficiently. The sheath composite was optimized taking into account the polymer choice based on the mechanical properties, conductive additive (carbon black) loading to achieve the best piezoresistive performance, and conditioning to stabilize the sensor’s signal. From the carbon black loading, the best performance was achieved with a high loading of 50 wt%, which was approximately 30 wt% higher than the percolation threshold. The sensor’s core material was varied to understand how the mechanical properties affect the sheath’s piezoresistive performance, and conditioning of the sensor was completed to stabilize the sensor’s signal. We tested the sensor using a random wave pattern over a 4-h period to mimic what a prototype device would be exposed to during use. We highlight the performance of the sensor for tracking strain using the piezoresistive signal by comparing root mean squared error calculations, and further improve these results by applying a machine learning algorithm to overcome any error in tracking that cannot be improved upon because of the limitations of the sensor to track efficiently in certain situations. We further explored the feasibility of using the optimized fiber strain sensor by developing a smart garment for measuring kinematic angles of the trunk relative to the pelvis in an exhaustive set of simple uniaxial and complex multiaxial movements. The design and development of this smart garment is described and, furthermore, the application of a machine learning algorithm to sensor signals for angle estimation is demonstrated. We evaluated the performance of this algorithm in a study with 12 participants performing simple to complex movements that are likely to occur in real application scenarios.

## 2. Materials and Methods

### 2.1. General Materials and Methods

Thermoplastic elastomer Hytrel 3078 (H3078) was obtained from DuPont (Kingston, ON, Canada). Polyether–urethane urea Dorlastane™ (PEU, 200 µm diameter multifilament) was bought from flaxerella.com. Carbon Black Vulcan XC72R (Cabot Corporation) was purchased from www.fuelcellstore.com. All other chemicals were obtained from Sigma-Aldrich and used as received. Tensile testing was completed on a linear stage built in-house (Figure S1). Scanning electron microscopy was completed on an Explorer 3 Desktop SEM sputter coated with 10 nm of iridium. Melt extrusion was completed using a Filabot EX2 Extrusion System. Two-point sheet conductivity testing was completed with a Fluke 115 True RMS Multimeter at 1-cm spacing.

### 2.2. Percolation Threshold for H3078 and Carbon Black

Thermoplastic Elastomer Hytrel 3078 (H3078) was first dissolved in CH_2_Cl_2_ at 5 weight% (wt%), and the appropriate amount of carbon black (0, 10, 15, 20, 30, 40, or 50 wt% wrt H3078) was then added and stirred for 30 min. The solutions were then cast onto glass slides, and put into a vacuum oven at 60 °C under reduced pressure for 30 min before conductivity testing was completed to ensure that any residual solvent was removed.

### 2.3. Sensor Production

#### 2.3.1. H3078 Coated with H3078:Carbon Black (50 wt%)

Thermoplastic Elastomer Hytrel 3078 (H3078) was extruded into a filament with a 0.4-mm die-producing filaments of ca. 450 µm, at a temperature of 190 °C, and the filament was allowed to spool 30 cm below the die. The filament was fed once through a solution of H3078 and carbon black (50 wt%) in dichloromethane (at a concentration of 5% w/v H3078) at a rate of 3.81 cm/second, and wound onto a bobbin 1.83 m from the exit of the coating solution (Figure 3d). Then, the bobbin containing the filament was put into a 60 °C vacuum oven under reduced pressure for 30 min to ensure that any residual solvent was removed. This produced a sensor with a sheath of 30 µm, as determined by scanning electron microscopy (SEM) (see Figure 3e,f).

#### 2.3.2. PEU Coated with H3078:Carbon Black (50 wt%)

Pristine PEU filament was fed once through a solution of H3078 and carbon black (50 wt%) in CH_2_Cl_2_ (at a concentration of 5% w/v H3078) at a rate of 3.81 cm/second and wound onto a bobbin 1.83 m from the exit of the coating solution. Then, the bobbin containing the wound filament was put into a 60 °C vacuum oven under reduced pressure for 30 min to ensure that any remaining solvent was removed. This produced a sensor with a sheath of 30 µm, as determined by scanning electron microscopy (SEM) (see Figure 3f).

### 2.4. Linear Stage (Tensile and Electronic) Setup and Testing

Tensile- and strain-resistance measurements were completed using a custom linear-stage with a Futek LFR400 (Futek Advanced Sensor Technology, Inc.; Irvine, CA, USA), connected to MATLAB (Figure S1). Testing was completed using either a sine, trapezoid, or random wave pattern (details below).

#### 2.4.1. Random Wave Pattern Testing

The random wave testing was completed by first creating a random series of waypoints, and then fitting a spline function to those waypoints, with a set of boundaries from 5–20% strain and a maximum strain rate of 15% s^−1^ for 1800 s (30 min). This was completed eight consecutive times with the same sensor to complete 4 h.

#### 2.4.2. Normalized Root Mean Squared Error (NRMSE) Analysis

Using software R and statistical package ‘pracma’, the position and resistance signals were normalized to values of 0–1 using:

(1) $$S{\left(t\right)}_{normalized}=\frac{S\left(t\right)-S_{\min}}{S_{max}-S_{\min}},$$

where S(t) is the signal value at time t, $S_{min}$ is the minimum value of that signal in each trial, $S_{max}$ is the maximum value of the signal, and $S{\left(t\right)}_{normalized}$ is the normalized value of the signal in time *t*. Then, the statistical analysis was completed to determine the normalized root mean squared error between the strain signal and resulting resistance signal. The result was multiplied by 100 to give a percent to one decimal place.

#### 2.4.3. Method for Random Forest Machine Learning Algorithm Analysis (RFMLA)

To further increase the accuracy of tracking the applied strain in random wave testing, a random forest machine learning algorithm (RFMLA) was implemented and trained using seven 30-minute blocks of random testing from 5–20% at 15% s^−1^. Random forest is an ensemble of decision trees each trained on a bootstrap sample of training data. Bootstrap samples are generated by randomly selecting data points from training data with replacement (i.e., one data point might be selected several times) [59]. Then, a decision tree is built using each bootstrap sample, and a subset of random features is used for splitting the nodes of trees. In comparison with a single decision tree, RFMLA is robust to overfitting on the training data and has a smaller generalization error [59]. Compared with other machine learning methods such as neural network and support vector regressor, RFMLA has shown better performance for processing strain sensor data in both regression and classification tasks [60,61,62]. The random forest model built-in Scikit-learn [63] package of Python was used in this study, and hyper-parameters were selected to achieve the highest accuracy. The number of trees was set to 100, and the maximum number of features used for splitting nodes was set to the root square of number of features. All other parameters were selected according to the default setting.

Using the sensor’s raw resistance as the input and the strain as the output of the model, a RFMLA model was trained, and its performance was evaluated by the 10-fold cross-validation method. Raw sensor data without any normalization was used as the input to the model, hypothesizing that RFMLA would find the correlation between the raw values of resistance and strain with better accuracy than the normalized RMSE analysis, and no pre-normalization is required when using this machine learning algorithm.

### 2.5. Method for Real Application Strain Range Calculation

**Figure 1:**
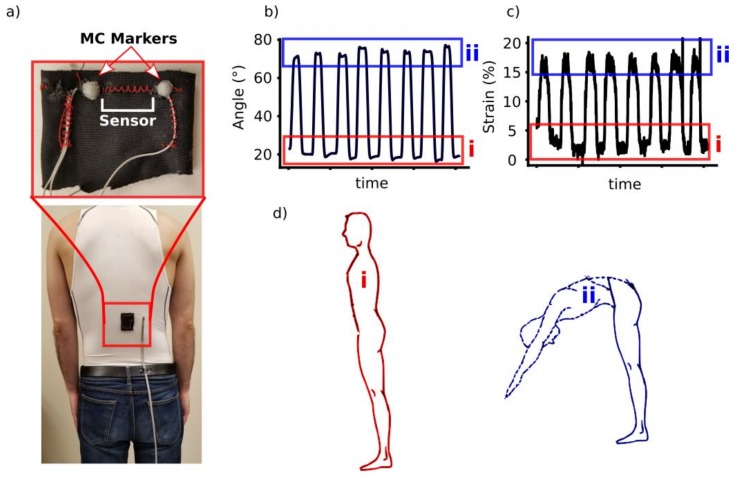
(**a**) A sample patch with motion capture markers fixed to an undershirt and worn to obtain the following values; (**b**) Angles obtained from a standing static position (i) to forward bending (ii); (**c**) Strain that is observed when going from a static standing position (i) to forward bending (ii); (**d**) static standing position (i) and forward bending (ii) completed for (**b**) and (**c**).

A motion capture system (Vicon, Oxford, UK) was used to record the position of markers on the body. Two markers were placed on the pelvis, and one marker was placed on the C7 vertebrae of a volunteer. Since the data collection was only for forward bending, using the three-marker angle of trunk relative to pelvis in the sagittal plane was calculated as the bending angle. Two other markers were placed at each end of the strain sensor stitched onto a piece of fabric, which was then attached to a stretchable undershirt (Figure 1a). The strain of the sensors was determined at two positions: (1) by standing straight—defined as pre-strain—and (2) forward bending (i.e., maximum strain, Figure 1b–d). Different locations on the back were tested to determine the best location for sensing. These motion capture markers were used to calculate strain of the sensor, which determined our working region of the sensor.

### 2.6. Smart Sensor Integrated Sleeveless Shirt

A sensor-integrated sleeveless shirt was developed to measure the three-dimensional angles of trunk movements relative to the pelvis. Our prototype was designed to be capable of measuring the trunk kinematics data in a variety of movements, including the uniaxial and multiaxial movements of the trunk. The following sections describe the elements of our design and further evaluate the prototype.

#### 2.6.1. Sensor Placement

**Figure 2:**
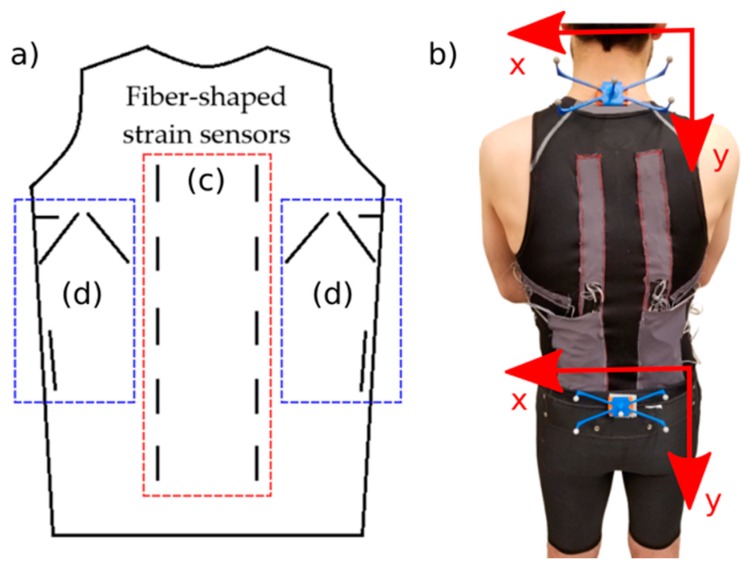
(**a**) Schematic illustration of 18 strain sensors placement on the garment. Each short line shows one strain sensor. Two vertical strips of sensors in the box (**c**) provide information for flexion, rotation, and lateral bending movements, whereas sensors in boxes (**d**) are specifically for detecting rotation and lateral bending, respectively. These sensors are isolated from trunk flexion movement. (**b**) The two-piece smart prototype with sensor integrated sleeveless shirt and shorts. Sensors were integrated on the back side of the shirt into the textile. The shirt was anchored to shorts with Velcro patches to minimize the upward shift at the waistline; two reflective marker sets were attached to the participant’s C7 and S1 vertebras for generating reference kinematic angles using the motion capture system.

The working principle of our smart garment was to monitor fabric strain that occurs because of the user’s motion. Mattmann et al. developed optical-based strain patterns of fabric on the trunk back side area while performing 27 distinct movements using a grid of reflective markers attached to the garment [64,65]. Within the patterns, there was consistent vertical strain along the length of the spine during trunk flexion–extension and lateral bending. Consmüller et al. used two strain gauge sensors strips attached to human skin on both sides of the spine, and using the information of two strips, showed that the upper body motions in different anatomical planes were distinguishable [66]. Consequently, we integrated two strips of sensors, each consisting of five 6-cm H3078 coated with H3078 strain sensors on both sides of the spine, covering the lower and upper back in a garment (Figure 2). Each sensor strip was 6 cm apart from the spine. In addition to the information that each one of these two sensor strips provided for detecting trunk flexion in forward bending, they can also be used for detecting movements in two other planes (lateral bending and rotation) by calculating the difference of the signals between the symmetrical sensors in the strips on either side of the spine. Using the strain patterns by Mattmann et al. as a starting point and conducting further empirical sensor placement tests, we found the placements shown in Figure 2 to provide the best strain patterns for trunk twisting and lateral bending [64,65]. This placement resulted in more sensitivity to rotation and lateral bending movements (as a result of more elongation of the sensors), which were isolated from the trunk flexion movement (no elongation of the sensors). In total, 18 sensors were integrated into the shirt for detecting all types of movements.

#### 2.6.2. Smart Sleeveless Shirt:

The sensor’s 450-µm diameter could allow the integration into textiles by stitching or weaving. This provided the potential to fabricate smart clothing with the sensors integrated directly into the textile structure. In our prototype, we attached the sensor to fabric using stitches over the length of the sensor (Figure 2). Wires were connected to the ends of the sensor using conductive ink and rubber glue. We used a commercially available tight-fitting sleeveless shirt for integrating the sensors. Eighteen sensors were integrated into the back side of the shirt to measure the strain pattern of the fabric. Shoulder and arm movements cause a significant unwanted strain pattern change in the fabric of sleeved shirts [37,64,65]. We intentionally chose a sleeveless shirt to reduce this unwanted change and isolate the strain pattern associated to trunk motions.

One crucial challenge of using strain-based clothing for trunk motion detection is the upward shifting of the garment at the waistline during movement. It has been shown previously that a 3-cm shift decreases the accuracy by more than 20% in an upper-body task classification problem using a smart catsuit [64,65], and care should be taken to minimize any waistline shift. The use of a leotard [67], catsuit [65], or straps [57,68] has been used to minimize this slippage. In an attempt to anchor the shirt to minimize this slippage, we opted to use a two-piece prototype (shirts and shorts) that allowed anchoring of the shirt to the shorts with Velcro patches. This allowed the comfortable extended use of this system under normal clothing without the use of a leotard/catsuit with minimal slippage. Figure 2 shows the two-piece prototype.

#### 2.6.3. Experimental Setup

A voltage divider was used for reading the change of resistance in sensors. Each sensor was connected to a resistor to form a voltage divider with a 5 V voltage source. The resistor value was selected to match the base resistance of the sensor, ca. 10 kΩ. Two data acquisition boards (Models NI BNC-2110 and NI BNC-2111, National Instruments, Austin, TX, USA) were used for reading the voltage signals of all the sensors. MATLAB R2017b (The MathWorks, Inc., Natick, MA, USA) was used for data collection.

For collecting the trunk kinematics data, we used a Vicon motion capture system (Vicon, Oxford, UK). This system consisted of six infrared motion tracking cameras. Two sets of reflective markers, each with five markers (8-mm diameter) were used to track objects. These two marker sets were mounted on the participants’ spinal C7 and S1 vertebras. The markers’ Cartesian coordinates information was used to generate the movements’ kinematic data. Figure 2b shows the tracker markers attached to a participant’s back. A synchronization signal from the motion tracking system was used for syncing the sensors and motion capture data. Data from all the components were recorded at a frequency of 100 Hz.

#### 2.6.4. Participants

To evaluate the performance of the prototype, 12 healthy male individuals were recruited between the ages of 25–35. The experimental protocol was approved by the Office of Research Ethics at Simon Fraser University. Prior to any data collection, written informed consent was obtained from all participants. Table 1 provides the characteristics of the participants.

#### 2.6.5. Study Protocol

The data collection for each participant lasted for 1.25 h, including the preparation time. The shirt was anchored to the short at the waist level using three Velcro patches. The positioning of the short was adjusted based on the participant’s feedback to ensure that the participants were comfortable during data collection. Then, the participants were asked to stand upright on a flat floor with feet shoulder-width apart and arms hanging to the sides of their body for the duration of the experiment. Then, a 10-s trial was recorded in this static posture. This trial was used for the standardization of all sensor data in the next trials.

The study protocol included four conditions of uniaxial movements and four conditions of multiaxial movements. Table 2 provides a list of the movements’ conditions. Since people’s common everyday movements include both uniaxial and multiaxial movements, an exhaustive combination of the uniaxial movements was selected as part of the study protocol. For each movement condition, participants were asked to perform the movement in three separate trials, each of them with the self-selected speeds of slow, moderate, and fast. In each trial, the movement was repeated 10 times. The range of motion of each repetition was self-selected by the participant, with a limit of maximum comfortable angle. This resulted in movements with different ranges of motion within each trial. For the random combination movement condition, participants were asked to perform random combinations of all the movements in a random order, with random ranges of motion for 60 s. During all the trials, the participants were asked to perform the movements with a natural manner and keep the self-selected speed constant.

#### 2.6.6. Reference Angle Measurement

Trunk reference kinematics angles (Eulerian roll, pitch, and yaw angles) were calculated using the motion capture (MoCap) data. Two marker sets were attached to the C7 and S1 vertebras. MoCap provides the Cartesian coordinates of the markers in each marker set. In each marker set, we constructed local unit vectors *X* and *Y* using the coordinates data and unit vector Z using the cross-product of *X* and *Y* unit vectors (Figure 2b). Then, the relative rotation matrix between C7 and S1 was computed:

(2) RS1C7=[XC7.XS1XC7.YS1XC7.ZS1YC7.XS1YC7.YS1YC7.ZS1ZC7.XS1ZC7.YS1ZC7.ZS1],

in which RS1C7 denotes the relative rotation matrix, and $X_{C_{7}},\;Y_{C_{7}},\;Z_{C_{7}}$ and $X_{S_{1}},\;Y_{S_{1}},\;Z_{S_{1}}$ are the $C_{7}$ and $S_{1}$ coordinate frame unit vectors, respectively. Each cell of this matrix was the inner product of the two vectors. Using this rotation matrix and the Z–Y–X Euler angles convention, Euler angles *ψ* (roll, flexion angle), *θ* (pitch, rotation angle) and *ϕ* (yaw, lateral bending) around the lateral–medial, superior–inferior, and anterior–posterior axes were calculated. The relative orientation of the trunk (C7) with respect to the pelvis (S1) was expressed using these three angles.

#### 2.6.7. Signal Processing

The voltage signals collected from strain sensors had a high-frequency noise. To remove this noise, we applied a 10^th^ order median filter to the voltage signal of each sensor. It has been reported that a 20-Hz frequency was a suitable data collection frequency for monitoring normal human activities [69]; consequently, the data voltage signals were then resampled to 20 Hz.

Slight differences between the sensors’ base resistance resulted in differences between the working voltage range of the sensors. Using the signal collected in the initial static trial, we normalized each sensor signal using:

(3) $$S_{i}{\left(t\right)}_{normalized}=\frac{S_{i}\left(t\right)-S_{i,mean}}{S_{i,max}},$$

where $S_{i}\left(t\right)$ is the sensor *i* voltage signal at time *t*, $S_{i,mean}$ is the mean value of that sensor voltage in the static trial, $S_{i,max}$ is the maximum value of the signal, and $S_{i}{\left(t\right)}_{normalized}$ is the normalized value of the signal in time *t*. Normalization brought all the signals from different sensors in the same working range.

As the placement of the sensors on the back side of the shirt was symmetrical, the difference between the signals of each symmetrical pair of sensors placed on the left and right sides of the shirt back was added to the raw data. ($∆v_{l}-∆v_{r})$ shows this difference, in which $∆v_{l}$ and $∆v_{r}$ are the left sensor signal and the corresponding symmetrical right sensor signal, respectively. In addition to this difference, we added the derivative of the signals to the raw data set. Adding these new signals provided the angle measurement algorithm with more information for detecting multiaxial movements and improved the angle estimation accuracy.

To generate the input for the angle measurement algorithm, a 1-s sliding window was used over the raw data signals. The window length was determined empirically by performing a grid search over windows with a length of 100 ms to 3 s to find the best performance in estimating the angles. Then, a feature extraction approach was applied to the raw signal data using the 1-s sliding window. The extracted features consisted of the minimum, maximum, mean, variance, median, root mean square, sum of absolute value, mean absolute deviation, wavelength, and slope sign changes. Then, the extracted features along with the raw data of the window were used as inputs for the angle measurement algorithm.

A random forest regressor algorithm was used for the angle estimation algorithm to train and test the model. Raw data along with the extracted features formed the input, and the roll, pitch, and yaw angles formed the output of the random forest regressor. The number of trees was set to 100, and the maximum number of features used for splitting nodes was set to the root square of number of features. All the other parameters were selected according to the default setting.

#### 2.6.8. Evaluation

The performance of the angle measurement machine learning algorithm was assessed by comparing the predicted angle from the algorithm with the reference true angle measured from the motion capture system. The coefficient of determination ($R^{2}$), the root mean squared error (RMSE), and the normalized root mean squared error (NRMSE) were used as the criteria for this comparison.

Using these criteria, we validated the performance of the machine learning algorithm in an intra-participant analysis. In this evaluation approach, one separate model was trained and tested for each participant. A three-fold cross-validation method was used to evaluate the performance of the model. Each fold comprised all the movement conditions with the same speed. Consequently, we had three folds corresponding to the slow, moderate, and fast movements. In this three-fold cross-validation approach, the model was trained using the data from two folds and tested on the remaining fold. This was repeated until all the three folds were selected as the test set. The accuracy of the model was determined by averaging the results of all three folds.

## 3. Results

### 3.1. Sensor Characterization

#### 3.1.1. Determining the Working Range of Desired Sensor

The maximum strain and pre-strain that the fiber sensor would be exposed to during use was analyzed using a motion capture system. This analysis determined that the sensor was strained approximately 5% when the garment was worn without any movement, which we have defined as pre-strain (Figure 1b,d). The portion of the lower back was identified that resulted in a maximum strain of approximately 20% strain. This correlated to angles of 20° for standing upright and 80° for forward bending, therefore resulting in a 60° range to track (Figure 1b,d). We defined the working range to be less than 30% strain for determining our sensor’s basic performance below, with the random wave pattern testing strain from 5–20% to mimic a sensor in a prototype device.

#### 3.1.2. Sensor Fabrication, Material Selection, and Basic Sensor Properties

**Figure 3:**
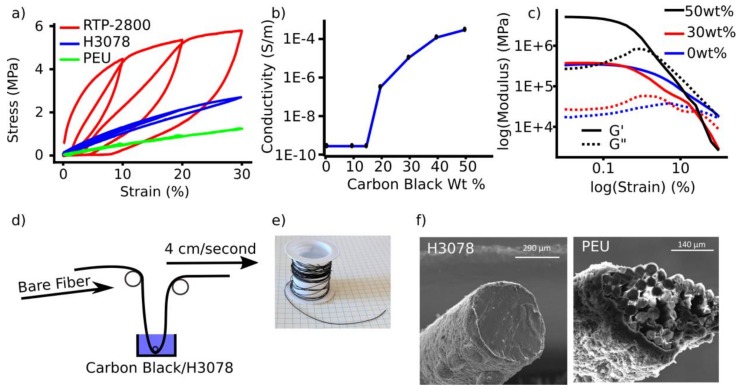
(**a**) Stress–strain curves for RTP-2800, H3078, and PEU; (**b**) Percolation threshold for carbon black in H3078; (**c**) Rheological amplitude sweep for carbon black loading in H3078; (**d**) Schematic for the dip-coating process of H3078 and PEU cores; (**e**) Resulting conductive fiber (H3078 core shown); (**f**) SEM image of core–sheath H3078 and PEU core sensors.

The mechanical properties of the filaments were first compared with stress–strain curves, and a cyclic hysteresis test in 10% increments up to 30% strain (minimal pre-load, ≤0.1 N). For comparison, a commercially available conductive thermoplastic elastomer, RTP-2800, was tested analogously. RTP-2800 showed large hysteresis, increasing with strain. RTP-2800 and H3078 had hysteresis values of 55% and 7% hysteresis at 10% strain; 61% and 8% at 20% strain; and 62% and 12% at 30% strain, respectively (Figure 3a) (Hysteresis Was Calculated by Analyzing the Area under the Curve and Completing a Percent Difference of That Area). Polyether urethane-urea (PEU, i.e., elastane/spandex) showed no hysteresis below 30% strain (Figure 3a).

The percolation threshold—which we have defined and empirically determined as the introduction of surface conductivity and piezoresistive properties on the sheath—was found to be 20 weight% carbon black in H3078 (Figure 3b). The effect of carbon black loading in H3078 on the mechanical properties was analyzed using rheological measurements. An amplitude sweep was completed at room temperature to determine the linear viscoelastic region, elastic (G’) and storage (G”) modulus, and the effect of carbon black addition (Figure 3c). H3078 had a storage modulus of 0.33 MPa, and a loss modulus of 0.03 MPa. With the addition of carbon black at 30 wt%, the storage modulus and loss modulus only slightly increased to 0.38 MPa and 0.03 MPa, respectively. The addition to 50 wt% carbon black significantly increased the storage and loss modulus to 5.15 MPa and 0.27 MPa, respectively. The linear viscoelastic region with respect to carbon black loading, and the rheological measurements indicated that the addition of carbon black decreased the viscoelastic region and stiffened the material substantially in comparison to the base polymer.

**Figure 4:**
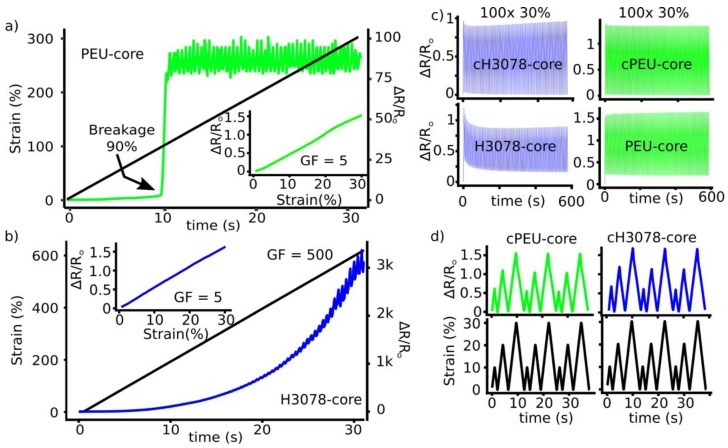
(**a**) PEU core sensor piezoresistive limit and sensor linearity up to 30% strain (inset); (**b**) H3078 core sensor piezoresitive limit and sensor linearity up to 30% strain (inset); (**c**) 100 repetitions of 0–30% strain for pristine H3078 core, PEU core, cH3078 core, and cPEU core sensors; (**d**) 10%, 20%, and 30% trapezoid strain for three cycles with cPEU core and cH0378 core sensors.

The sensors were initially analyzed by completing strain-resistance measurements to determine the signal quality and accuracy within our desired working range (<30% strain). Sheaths produced from 30 weight% carbon black resulted in signals that suffered from hysteresis, drift, and did not track strain accurately (Figure S2). Increasing carbon black content to 50 wt% improved the linear piezoresistive signal tracking (Figure S2). H3078 core sensors suffered from the first cycle hysteresis and signal drift, whereas the first cycle hysteresis of PEU core sensors was less, and had a more consistent signal over 100 cycles at 30% strain (Figure 4c, bottom graphs). Conditioning the sensors (discussed below, labeled cH3078 and cPEU) removed the first cycle hysteresis and improved the consistency of the signal over 100 cycles. The gauge factor of the H3078:CB sheath at 30, 40, and 50 wt% loading was 3, 4, and 5 for up to 30% strain, respectively (Figure S2).

#### 3.1.3. Strain Limit of Sensors

The strain limit of the sensor was determined using a strain ramp experiment. A loss of piezoresistance was marked by a large increase in resistance. This data was important to identify: (1) any linear resistance–strain regions; (2) the resulting gauge factor; and (3) the strain limit of the sensing capabilities of the sensor. The H3078 core sensor was able to access large strains, upwards of 600% before a loss of piezoresistance and a maximum gauge factor of 500 (Figure 4b). The PEU core sensor was only able to undergo strains of up to 90% before a loss of piezoresistance (Figure 4b). The PEU core sensor had noticeable cracking of the H3078 carbon black sheath beyond 90% strain, which was likely a result of poor adhesion from the PEU core to the conductive H3078 carbon black sheath.

#### 3.1.4. Sensor Conditioning

As previously noted, H3078 core and PEU core sensors underwent first cycle drift upon straining to 30% with new pristine sensors. The H3078 core sensor signal started to stabilize over 100 cycles, while the PEU core sensor was fairly consistent over 100 cycles. To overcome both the first cycle hysteresis and resistance drift, both the H3078 core and PEU core sensors were conditioned above the intended working range of 30% strain. This was completed at 40%, 80%, and 120% strain for 100 cycles using a sinusoidal wave pattern at a strain rate of 10% s^−1^. Since the PEU core sensors had a limit of 80–90% strain, this was only completed at 40% for those samples. The sheaths of H3078 core sensors were imaged using scanning electron microscopy to observe any obvious damage to the sheath. The conditioning at 80% and 120% strain produced larger cracking and gaps in the conductive sheath, which were not observed in the pristine and 40% conditioned samples (Figure S3). The 80% and 120% conditioned samples also suffered from lower performance, and therefore, the 40% conditioning was chosen for more in-depth testing: these were labeled the cH3078 core and cPEU core sensors. The stability of the piezoresistive signal of cH0378 cores and cPEU cores were tested analogously to pristine sensors over 100 cycles from 0–30% strain, resulting in no first cycle hysteresis and a consistent signal (Figure 3c).

#### 3.1.5. Sensor Linearity

Ideal piezoresistive sensors possess a stable and linear relationship between resistance and strain. This requires a consistent gauge factor within the working range. After conditioning the H3078 and PEU core sensors (cH3078 core and cPEU core), both sensors had ideal linear relationships within the working range of 30% strain (Figure 4a,b inset graphs). Furthermore, both sensors had little first cycle drift, and could be repetitively cycled to 10%, 20%, and 30% strain without signal drift or a change in gauge factor (Figure 4d).

#### 3.1.6. Effect of Strain Rate on Sensor Performance

The effect of strain rate on the sensors was tested by completing a simple 0–10% sinusoidal wave pattern while varying only the strain rate. This was completed at a higher frequency (1 Hz) and lower frequency of (0.1 Hz) for both cH3078 and cPEU core sensors (Figure S4). Both cH3078 and cPEU core sensors suffer from a small amount of rate-dependent piezoresistive properties at 1 Hz, as indicated by the decreasing piezoresistive signal versus that of one that is consistent at the slower strain rate of 0.1 Hz [70,71,72].

#### 3.1.7. Sensor Testing: Random Wave Pattern to Simulate Real Events

**Figure 5:**
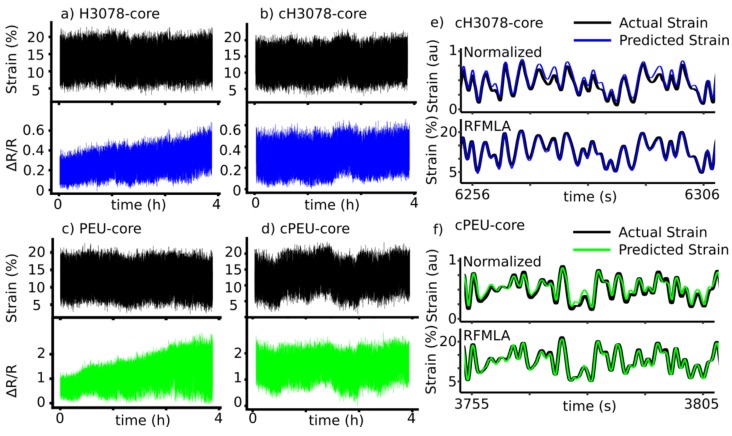
Random wave testing between 5–20% strain (top, black) and resistance signal (bottom) for (**a**) an H3078 core sensor (blue), (**b**) a cH3078 core sensor (blue), (**c**) a PEU-core (green), and (**d**) a cPEU core sensor (green); (**e**) Snapshot overlay of predicted strain using normalized resistance signals (top) and random forest machine learning algorithm (RFMLA) predicted strain (bottom) for the random wave testing for cH3078 core sensors; (**f**) Snapshot overlay of predicted strain using normalized resistance signals (top) and RFMLA predicted strain (bottom) for the random wave testing for cPEU core sensors.

With both the pristine and conditioned sensors (H3078 core, PEU core, cH3078 core, and cPEU core, respectively) we sought a methodology that would allow us to mimic the conditions that the sensors would be exposed to in prototype devices. The methodology employed utilizing the known working range of the sensor for our specific application, which we defined (5–20% strain) with a strain rate up to 15% s^−1^. Random wave pattern testing was completed in 30-min intervals, for a total of 4 h on both non-conditioned (H3078 core and PEU core sensors) and conditioned (cH3078 core and cPEU core sensors). Empirically, the average resistance values of H3078 core sensors increased over 4 h (Figure 5a). The PEU core sensors gauge factor was increasing over the 4 h, although the baseline resistance stayed consistent (Figure 5c). The cH3078 core and cPEU core sensors did not have any of the noticeable signal drift or change of baseline that was observed in non-conditioned samples (Figure 5b,d).

Analysis of these 4-h random wave pattern test experiments was completed initially by two simple methods. First, each 30-min block was normalized, and the accuracy of the sensor was determined using a normalized root mean square error (NRMSE) calculation (see the Methods section for details). The second method involved concatenating the 30-min blocks, followed by NRMSE calculations. The second of these two methods highlighted the variability of the sensor signal throughout the 30-min blocks (which was visually apparent in Figure 5a,c, as discussed above). This gave an indication of the consistency of the sensor over longer periods of testing. Pristine H3078 core sensors had an average NRMSE of 7.7% when normalized within the 30-min testing intervals, and increased to 15% when normalized as a four-hour testing block (see Table 3 for all NRMSE values). Pristine PEU core sensors had an NRMSE of 9.3% when normalized within the 30-min testing intervals, and increased to an NRMSE of 16% when normalized as a 4-h block. The cPEU core NRMSE in the 30-min normalization calculations decreased to 5.0% from 9.3%, while the cH3078 core sensors decreased to 7.1%. Normalizing the concatenated 4-h testing block, the cPEU core sensor increased in performance to an NRMSE of 6.3% from 16%, and the cH3078 core sensor improved to 6.4% from 15%.

#### 3.1.8. Sensor Optimization Using Machine Learning Algorithms

With the raw data resulting in NRMSE values as low as 5%, we sought to further increase the accuracy of the sensors’ tracking of strain using the piezoresistance by training a random forest machine learning algorithm (RFMLA) using seven 30-minute blocks of random testing and analyzing a separate 30-min testing block. RFMLA has shown promising performance in the regression on strain sensor’s data for our prototypes [60,61,62]. The use of RFMLA improved the tracking of the strain with cH3078 core sensors to a NRMSE of 1.6%, while the cPEU core sensors improved to 2.6%.

### 3.2. Smart Sleeveless Shirt Testing

For each participant, one-hour trunk motion data comprised of 24 different trials was collected. In each movement type, only the relevant sensors of that movement type were strained so that the voltage patterns were different between different movement types (Figure S5). Averaged across all uniaxial and multiaxial movement conditions, the random forest regressor estimated the roll angle with $R^{2}$ = 0.94 ± 0.02, RMSE = 4.26° ± 0.73°, and normalized RMSE (NRMSE) = 5.14% ± 0.64%; the pitch angle with $R^{2}$ = 0.92 ± 0.02, RMSE = 3.53° ± 0.52°, and NRMSE = 4.68% ± 0.58%; and the yaw angle with $R^{2}$ = 0.91 ± 0.03, RMSE = 3.44° ± 0.52°, and NRMSE = 5.79% ± 0.73% averaged over all participants (Table S1 for the details of all participants). Table 4 reports the detailed performance of the algorithm for each of the three self-selected speeds. Among the three angles, movements with moderate speed had the best performance with the maximum accuracy ($R^{2}$ = 0.96 in flexion angle), whereas fast movements had lower angle estimation accuracies ($R^{2}$ = 0.90 in rotation angle).

Table 5 presents the detailed results of angle estimation in each movement condition among all participants. The algorithm estimated the principal angles in uniaxial movements accurately ($R^{2}$ > 0.97). As the movement became more complex, the error in estimating angles increased with the maximum error happening in the random combination movement condition (NRMSE between 7%–10%).

**Figure 6:**
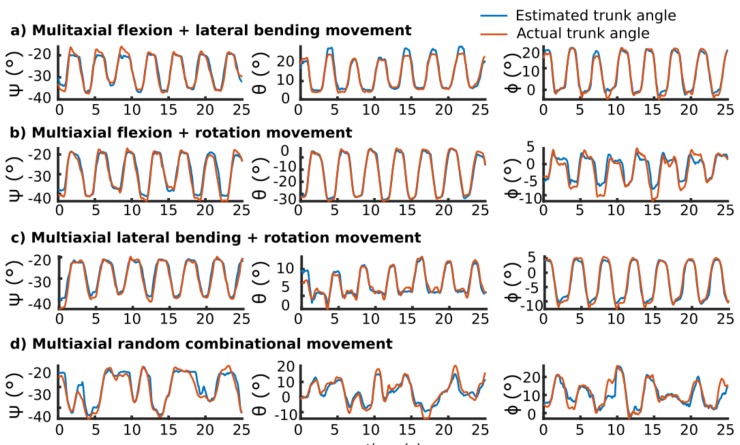
Exemplary comparison between three kinematic reference and estimated ψ (flexion), θ (rotation), and ϕ (lateral bending) angles in multiaxial movements. Most estimation errors happened at peaks where the algorithm cannot estimate the exact value. (**a**) Flexion and lateral bending movement; (**b**) Flexion and rotation movement; (**c**) Lateral bending and rotation movement; and (**d**) Multiaxial random combination movement.

Figure 6 demonstrates all three angles in four multiaxial movement conditions (see Figure S6 for an example of uniaxial movement conditions). Random forest regressor estimation follows the actual angle pattern in all complex movements ($R^{2}$ > 0.82). The flexion angle had the minimum decrease in accuracy with $R^{2}$ = 0.85 in the random combination movement condition.

## 4. Discussion

We hypothesized that creating a fiber sensor with a supportive non-conductive elastic core covered with a conductive sheath would improve the common problems associated with conductive composite sensors—such as a decrease in viscoelastic region and stiffening of the polymer. Piezoresistive sensors rely on the electrical disconnections of conductive additives within the polymer matrix upon the application of strain [52]. If plastic deformation occurs, the baseline resistance values and gauge factors will change. This makes using the piezoresistive signal difficult for tracking strain accurately. A core–sheath morphology could provide the required support for the piezoresistive composites in a standalone morphology that could be truly integrated seamlessly into textile base wearable devices.

Multidisciplinary research combining both sensor design, fabrication, and optimization; subsequent use in prototype devices for use in larger cohort kinematic tracking studies has yet to be reported. Our goal for this report was to fabricate a sensor that:

(1) Could be made from readily available materials.
(2) Would be reliable and accurate within the intended working range for our application.
(3) Would be accurate at tracking strain when exposed to random movements that are typical in prototype devices over longer testing periods.

The performance of sensors has been improving dramatically with recent reports accessing strains upwards of 1000% [36,53], large gauge factors exceeding 10^6^ [73], and resistance–strain linearity beyond 120% strain [36]. A more in-depth larger cohort testing for the accuracy and feasibility of these sensors for three-dimensional kinematic movement has been much less common, and most sensor reports have limited proof-of-concept results. Furthermore, our aim to provide a methodology to test sensors in a way that mimics prototype devices would allow a rigorous analysis of future sensors prior to any larger cohort prototype device testing, resulting in better performance with more accurate kinematic data for analysis.

To begin the fabrication of the sensors, we explored a variety of conductive additives, but chose a high conductive grade of carbon black. This was simply the lowest cost conductive additive requiring no specialized mixing methods such as sonication or shear mixing when using polymer solutions. H3078, a thermoplastic elastomer with low hysteresis within the desired working range (<30% strain, Figure 3a), required 20 weight% carbon black to reach the percolation threshold, but had the best performance at 50 weight% loading. This high loading of carbon black affects the mechanical performance of the material by increasing both the elastic and storage modulus of the material and lowering the viscoelastic region of the material (Figure 3c). This unfortunately decreased the ideal elastic properties of the material, and would result in more plastic deformation of the composite if used independently as a material for a fiber sensor. This would cause a detrimental piezoresistive baseline signal drift because of the lack of reconnections within the conductive pathways of the composite after plastic deformation. Notably, these have been used previously, but with a supporting elastic covering to reduce large plastic deformations [37,57,58]. We hypothesized that including an elastic core would support a piezoresistive sheath—within the core’s elastic region—and decrease the amount of problematic piezoresistive signal baseline drift, since the sheath would return to its original length, and the core would be dominating the mechanical properties over the thinner sheath layer. Two core materials were chosen: A thermoplastic elastomer H3078—the same material used for the piezoresistive sheath—and PEU—otherwise known as spandex—a ubiquitous highly elastic, low hysteresis polymer.

Applying the conductive composite to the cores was completed using a one-step dip-coating process. The advantage of using a piezoresistive polymer composite sheath was the ease of application, requiring only one dip-coating step. Typically with graphene or carbon nanotube coatings, multiple coatings or sequences are required to deposit enough conductive material to create a piezoresistive layer [36,54]. This technique is attractive, since it could be implemented easily as a finishing step during fiber production using a roll-to-roll manufacturing process such as those used for wet spinning, wet–dry spinning, melt spinning, or electrospinning [74].

The accessible piezoresistive sensing strain of the H3078 core sensors was higher compared to the PEU core sensors. The PEU fiber was more elastic in comparison to H3078 fibers beyond 30%, and could withstand large strains beyond 1000%, but the adhesion between the core and sheath were such that the sheath underwent irreversible damage beyond 90% strain, causing a loss of signal. There was also noticeable cracking in the sheath of the PEU core sensor beyond 90%. Some sensors that are produced from pure conductive materials such as carbon nanotube forests or Ag nanoparticles rely on cracking for piezoresistance [56,73,75]; however, for piezoresistive composites, this is a point of failure. It was concluded that the H3078 core fibers’ advantage of using a one-step dip-coating process with a solvent that softens/dissolves a thin layer of the outer portion of the core allowed better adhesion of the sheath to the core by increasing polymer entanglement at the interface [76]. Producing PEU core sensors with a PEU-based sheath could have improved the strain limit of the sensor, although this was not explored, since PEU was only soluble in high boiling point solvents (such as N,N-dimethyl formamide or N-methyl-2-pyrrolidone) that did not permit coating with an analogous procedure.

Both the H3078 core and PEU core sensors required conditioning (stretching the sensor repetitively to a specific strain) prior to use. It was observed that H3078 core sensors had large first cycle hysteresis and a signal drift that stabilized over 100 cycles at 30% strain (Figure 4d). PEU core sensors did not suffer from as large a first cycle hysteresis, and had a consistent signal throughout 100 cycles at 30% strain (Figure 4d). It was hypothesized that in H3078 core sensors, both the core and sheath were undergoing some plastic deformation in these initial tests, and the sheath was undergoing conductive pathway destruction [73,77]. The PEU of the PEU core sensors were not undergoing plastic deformation—which would explain the smaller first cycle hysteresis, since the sheath material would return to its original length—and the stability of the signal was likely directly related to the higher elasticity and low hysteresis of the PEU core. The sheath was able to return to its original length because of the elasticity of the PEU core, and therefore reconnect the majority of conductive pathways, minimizing signal drift. To eradicate any piezoresistive signal drift and first cycle hysteresis, both sensors were conditioned above the working range of 30% to strains of 40%, 80%, and 120% for 100 repetitions. PEU core sensors were only able to undergo conditioning at 40% strain. SEM imaging of the conditioned sensors revealed larger cracking in the sheath for the 80% and 120% conditioned sensors (Figure S3). Furthermore, sensors conditioned at 80% and 120% decreased in performance, and were therefore discarded from further testing. Conditioning to 40% strain did not noticeably affect the sheath’s morphology by SEM, and improved the performance such that we were able to track 10%, 20%, and 30% strains repetitively and accurately with a stable baseline with both cH3078 and cPEU core sensors (conditioned sensors, Figure 4f). These sensors also had a linear correlation for a change in resistance to strain up to 30% with a gauge factor of five (Figure 4e). Recent reports of core–sheath fiber strain sensors have similar gauge factors at low strain [44,54], while most have strived to achieve high gauge factors at larger strains that are not required for this type of application.

Finally, for sensor characterization, we explored the limitations of the sensor. The high strain limit of the H3078 core sensor was 600% with a maximum gauge factor of 500, while PEU core sensors were only able to access 90% strain. Therefore, the sensors were well above the required strain to perform in our intended application. Strain rate dependence was explored using two frequencies of 0.1 and 1 Hz (2% s^−1^ and 20% s^−1^, respectively). The piezoresistive signal underwent a small amount of rate-dependent hysteresis and signal compression at the higher 1-Hz strain rate (Figure S4) [71,72,73]. The slower strain rate of 0.1 Hz did not show this phenomenon. Piezoresistive sensors have been reported that are able to track at higher strain rates, although they do not utilize polymer composites and rely on crack formation and decreased contact as the mechanism of piezoresistivity, avoiding this effect from the polymer mechanical properties [78]. If fast strain rates are required, the use of capacitive sensors are more appropriate, since they do not depend on the piezoresistive mechanism susceptible to rate-dependent hysteresis, and can sense accurately at strain rates upwards of 50% s^−1^ [79]. This limitation of the sensor is directly related to the inherent material properties, yet for signal analysis, this information is useful. These limitations may indicate the type of movements and scenarios in prototype testing that result in lower accuracy. Furthermore, if the rate-dependent signal effects are predictable, analysis using machine learning algorithms may be capable of predicting the actual strain profile.

With cH3078 core and cPEU core sensors in hand, we created a testing protocol that would allow us to monitor both tracking accuracy within our defined working region (<30%, specifically 5–20%) and the effect of random movements that are likely to occur during a larger cohort prototype testing of a wearable device with these sensors. We sought a testing protocol that would mimic prototype testing conditions to first understand how the sensors behave in different conditions, and how accurate we could get to predicting the strain of the sensors when exposed to random motion that would be seen in prototype devices. Using the results of this testing protocol, we further used the optimized cH3078 core sensor to develop a smart textile garment that is capable of tracking three-dimensional random back movements, and evaluated the performance of the sensor in a prototype device for tracking movements that are most likely to happen in real applications of the sensor. Thus far, this type of testing to mimic a prototype device to gauge performance has not been reported.

The 4-hour testing blocks were completed to mimic the constant change of strain and strain rate that prototype devices would be expected to track over a day of use. We tracked H3078, PEU, cH3078, and cPEU core sensors to further show the benefit of conditioning the sensors prior to use (Figure 5a–d). With conditioning, both the cH3078 and cPEU core sensors had a stable resistance signal over the 4-h testing periods, which was highlighted by the sharp drop in NRMSE when calculated by concatenating the signals over the full 4-h with NRMSEs at 6.4% and 6.3%, respectively (Figure 5c,d, Table 3). These results are unique, since we obtained a performance metric from a test that is likely more demanding than what a normal person would expose these sensors to, with accuracies similar to those of other wearable technologies [80].

While this result was promising, we have previously reported the use of machine learning to increase accuracy in sensor tracking after normalization [60]. We hypothesized that using machine learning algorithms could improve the tracking if the events causing tracking errors were reproducible and consistent during the random wave pattern testing. Reducing the amount of signal processing would also be advantageous in prototype devices; therefore, we tested the accuracy of RFMLA without any signal normalization. Raw signals analyzed by the RFMLA resulted in the lowest NRMSE values of 1.6% and 2.6% for cH3078 and cPEU core sensors. This indicated that the sensors were in fact responding reproducibly and consistently throughout our 4-h testing period, and the RFMLA was able to adjust the predicted strain to higher accuracy. These results indicate that this sensor would be capable of measuring the angle of the lower back in complicated multiplanar movements. However, careful design of prototypes considering optimized sensor placement, modified signal preprocessing, and specific data analysis approaches are still required to achieve promising results in tracking the angles of three-dimensional movements.

Developing wearable motion capture systems for unobtrusive daily use is an ongoing challenge. In this work, we developed a smart sleeveless shirt by integrating the cH3078 core strain sensors into a commercially available garment. We investigated the feasibility of this smart garment in capturing all types of planar uniaxial and complicated multiaxial trunk motions.

The results of our work showed a high agreement between the estimated angles using the smart garment and the angles calculated from the motion capture system. All three angles were estimated with an RMSE of less than 4.26° averaged over all movement conditions. Cuesta et al. suggested that an error between 2–5° is likely to be regarded as an acceptable accuracy for wearable systems, while extra consideration is required for clinical applications [80]. The accuracy of our system fell within this range, and would be sufficient for a wearable motion tracking system. Furthermore, there was a high correlation between the estimated angle and real angle, with an $R^{2}$ higher than 0.91 for all angles. Similar to other systems, the correlation of estimate to real angles was affected by the movement complexity [81]. Our results showed that in simple planar movements, the estimation followed the real pattern accurately ($R^{2}$ > 0.97). As the movement became more complex, the level of agreement between the estimated and real angles decreased ($R^{2}$ > 0.83), but the RMSE error stayed below 5°.

The random forest regressor algorithm estimated the trunk flexion, rotation, and lateral bending with RMSEs of 4.26°, 3.53°, and 3.44°, respectively in our system. IMU-based motion captures are the most common wearable motion captures. Several previous studies have investigated the validity of IMU-based systems for motion analysis [26,27]. Schall et al. reported an accuracy of 4.1° to 6.6° for trunk motion monitoring during a field-based study [82]. Samadani et al. reported errors less than 2° for trunk planar angle measurement [30]. Our results compare well to the field-based study, but they fall lower than the results of Samadani et al. It should be emphasized that the data collection protocol in the Samadani et al. report was simple, including only five repetitions of planar movements, while in other studies with more complicated protocol, an error between 2–5° was reported for these IMU-based systems [80]. Considering IMUs’ limitations of susceptibility to magnetic disturbances and drifting over time [83,84], our developed smart shirt is comparable to IMU-based systems and superior for environments where magnetic distortions are present. This is the case for most environments in which ferromagnetic materials such as metals are found.

To the best of our knowledge, there have been limited studies investigating the feasibility of smart garments based on textile-based sensors measuring angles during multiaxial trunk motions. Mokhlespour et al. developed a smart undershirt that was capable of measuring planar lumbar movements with 1.3° error when measuring single degree-of-freedom movements [57]. Therefore, no conclusions could be drawn about the application of their system to daily usage where multiaxial movements are inevitable. Mattmann et al. developed a smart catsuit using similar sensors for task classification, although no angle measurement was completed [64,65]. Yamamoto et al. used stretch sensors fixed to the skin for measuring complicated lumbar motion angles [58]. Complicated movements resulted in errors higher than 10° in measuring trunk flexion–extension angles. Our study showed superior results in measuring trunk three-dimensional angles while performing complicated movements.

Among the three estimated kinematic angles, the pitch angle (corresponding to trunk rotation) was estimated with 4.68% error. It has been previously shown that the most challenging angle to measure is the trunk rotation angle [85]. Measuring trunk rotation angles using IMU-based systems has been difficult because of the susceptibility of IMU sensors to magnetic distortion [31,83], which is a problem where textile-based sensors could provide an alternative, accurate solution.

The accuracy of the prototype device did have dependence on the speed of movements, although there was only minimal increase in error during fast movements by only 1.5° for flexion, and 1° for rotation and lateral bending.

Overall, our developed smart tank top system was able to track multiaxial trunk movements in three dimensions with an error less than 4.26°. This shows the great potential of smart textile systems to be used as wearable motion tracking systems. This will provide us with tools for long-term unobtrusive data collection from human movements. This data could supply useful information for applications such as developing personalized interventions to decrease the occurrence of lower back pain among health care workers.

## 5. Conclusions

A core–sheath piezoresistive sensor was produced from readily available materials without the use of specialized mixing or processing requirements. A specific application was chosen to determine the working range of the intended fiber sensor (<30% strain). Then, sensors were produced by one dip-coating procedure with varying amounts of conductive carbon black to determine the percolation threshold and the performance with respect to carbon black loading. The 50 wt% carbon black performed significantly better than lower carbon black loadings with respect to linear response and hysteresis. The piezoresistive sheath was applied to two cores with different mechanical properties to determine the supportive core’s effect on the sensor’s performance. Better adhesion of the sheath to an H3078 core allowed higher accessible sensing limits, while the PEU core sensor had lower first cycle hysteresis and a more stable resistance signal. Conditioning the sensors to 40% strain (10% above the intended working region) resulted in sensors without first cycle hysteresis and a stable baseline resistance. To determine the sensor’s performance prior to use in prototype devices intended for accurate three-dimensional kinematic movement tracking, they were analyzed using a random wave pattern on a linear stage. We were able to track strain with the piezoresistance signal to a NRMSE of 6.3% with conditioned sensors using a simple normalization procedure. While we were aware of the limitations of the sensor with respect to rate-dependent behavior causing signal compression, we were able to overcome the inaccuracies of the sensor by utilizing machine learning algorithms to decrease the error of the strain tracking to 1.6% NRMSE. Then, we developed a smart textile system using the H3078 core fiber strain sensor for measuring trunk three-dimensional kinematic angles. Using random forest regressor, this system detected trunk flexion, rotation, and lateral bending angles with 4.26°, 3.52°, and 3.4° errors, respectively. This showed the feasibility of a smart textile system using our developed sensor for measuring the kinematic data of complicated multiaxial movements. This system could be useful for developing alternatives for the standard kinematic motion-tracking devices composed of IMUs and motion-capture devices that are bulky, non-portable, spatially limited, and susceptible to external magnetic distortions.

## Figures and Tables

**Table 1:** Participants characteristics.

Categories	Participant
Age (years)	28 (3.3)
Height (cm)	177 (7.6)
Weight (kg)	75 (9.8)

**Table 2:** Study protocol movement conditions and types.

Trial Number	Movement Condition	Movement Type
1–3	Rotation	Uniaxial
4–6	Lateral Bending (LB)	Uniaxial
7–9	Flexion	Uniaxial
10–12	Slouching	Uniaxial
13–15	Flexion + Lateral Bending	Multiaxial
16–18	Flexion + Rotation	Multiaxial
19–21	Lateral Bending + Rotation	Multiaxial
22–24	Random Combination	Multiaxial

**Table 3:** Normalized root mean squared error (NRMSE) data for H3078 core and PEU core sensors with different analysis procedures.

Sample	NRMSE ^a^	NRMSE ^b^	RFMLA NRMSE ^c^
H3078 core	7.7%	15%	-
cH3078 core	7.1%	6.4%	1.6%
PEU core	9.3%	16%	-
cPEU core	5.0%	6.3%	2.6%

^a^ Normalized each 30-min section. ^b^ Normalized over complete 4-h testing period. ^c^ No normalization completed before RFMLA analysis.

**Table 4:** Performance results of the algorithm in the detection of three angles of ψ (flexion), θ (rotation), and ϕ (lateral bending) for different speeds averaged across all participants. NRMSE: normalized RMSE.

	Ψ			θ			ϕ		
Speed	$R^{2}$	RMSE (Deg)	NRMSE (%)	$R^{2}$	RMSE (Deg)	NRMSE (%)	$R^{2}$	RMSE (Deg)	NRMSE (%)
Slow	0.94	4.12	5.05	0.93	3.35	4.70	0.92	3.26	5.73
Moderate	0.96	3.61	4.55	0.94	3.12	4.09	0.93	3.03	5.10
Fast	0.92	5.06	5.82	0.90	4.12	5.25	0.88	4.02	6.55
Average	0.94(0.02)	4.26(0.73)	5.14(0.64)	0.92(0.02)	3.53(0.52)	4.68(0.58)	0.91(0.03)	3.44(0.52)	5.79(0.73)

**Table 5:** Performance results of the algorithm in the detection of three angles of ψ (flexion), θ (rotation), and ϕ (lateral bending) for each movement condition. NRMSE: normalized RMSE.

	ψ			θ			ϕ		
Movement	$R^{2}$	RMSE (deg)	NRMSE (%)	$R^{2}$	RMSE (deg)	NRMSE (%)	$R^{2}$	RMSE (deg)	NRMSE (%)
Rotation	0.94	3.23	3.91	0.97	1.66	2.41	0.94	2.45	3.73
Lateral Bending	0.93	3.65	4.32	0.83	3.60	3.07	0.98	1.94	2.84
Flexion	0.97	3.08	3.49	0.85	4.16	3.52	0.87	3.13	4.34
Slouching	0.95	2.39	3.39	0.93	1.17	2.85	0.94	2.12	4.26
Flexion + Lateral Bending	0.87	4.27	8.78	0.89	4.08	4.85	0.92	3.12	6.04
Flexion + Rotation	0.92	4.60	7.10	0.87	4.36	6.83	0.82	3.02	8.03
Lateral Bending + Rotation	0.92	4.29	7.25	0.86	4.26	5.83	0.97	3.38	5.42
Random Combination	0.85	5.13	7.74	0.83	4.90	10.67	0.85	4.48	10.09

## Supplementary Materials

The following are available online at https://www.mdpi.com/1424-8220/19/19/4288/s1, Figure S1: Linear stage set up; Figure S2: 30, 40, and 50 wt% carbon black loading in J3078 trapezoidal wave pattern at 10, 20, and 30% strain.; Figure S3: SEM images of 40, 80, and 120% strain profiles for H3078 sensors; Figure S4: Frequency dependent behavior of H3078-core sensors strain from 0-10% using a sinusoidal wave pattern A) 1Hx, B) 0.1 Hz; Figure S5: Unnormalized change of strain sensors voltage while performing 3 different types of movement; Table S1: Performance results of the algorithm in the detection of 3 angles of flexion, rotatation, and lateral bending for each participant; Figure S6: Exemplary comparison between the principle reference and estimated flexion, rotation, and lateral bending angles in uniaxial movements.
