# Supplementary material for: Application-Based Production and Testing of a Core–Sheath Fiber Strain Sensor for Wearable Electronics: Feasibility Study of Using the Sensors in Measuring Tri-Axial Trunk Motion Angles
Source: Sensors (Basel). 2019 Oct 3;19(19):4288. doi: 10.3390/s19194288 (PMC6806223; doi:10.3390/s19194288)
Supplement: Supplementary file 1 [file sensors-19-04288-s001.pdf]

Supporting Information for:

Application-Based Production and Testing of a Core-Sheath Fiber Strain Sensor for Wearable Electronics: Feasibility Study of using the Sensors in Measuring Tri-Axial Trunk Motion Angles

Ahmad Rezaei, Tyler J. Cuthbert, Mohsen Gholami and Carlo Menon*

Figure 1. Linear stage set up.

Figure S2. 30, 40, and 50 wt% carbon black loading in H3078 trapezoidal wave pattern at 10, 20, and 30% strain

Figure S3. SEM images of 40, 80, and 120% strain profiles for H3078 sensors.

Figure S4. Frequency dependent behaviour of H3078-core sensors strain from 0-10% using a sinusoidal wave pattern A) 1 Hz; B) 0.1 Hz

Figure S5. Unnormalized change of strain sensors voltage while performing 3 different types of movement. As a result of the specific sensor placement, voltage change depended on movement type and in each specific movement, only sensors related to that movement were strained. (a) Uniaxial flexion movement of the trunk; (b) Uniaxial rotation movement of the trunk; (c) Uniaxial lateral bending of the trunk.

Table S1. Performance results of the algorithm in the detection of 3 angles of ψ (flexion), θ (rotation), and ϕ (lateral bending) for each participant (P).

Figure S6. Exemplary comparison between the principal reference and estimated ψ (flexion), θ (rotation), and ϕ (lateral bending) angles in uniaxial movements. (a) Uniaxial flexion movement; (b) Uniaxial rotation movement; (c) Uniaxial lateral bending movement.

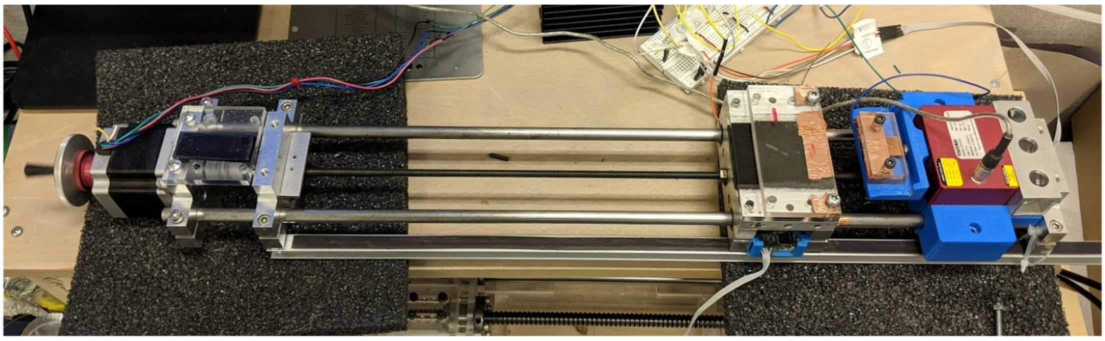

Figure S1. Linear stage set-up for fiber strain sensor testing.

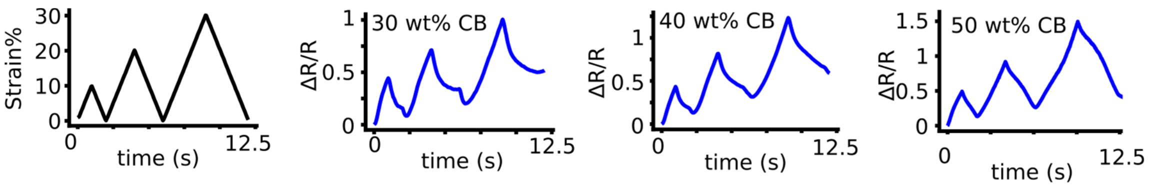

Figure S2. 30, 40, and 50 wt% carbon black loading in H3078 trapezoidal wave pattern at 10, 20, and 30% strain.

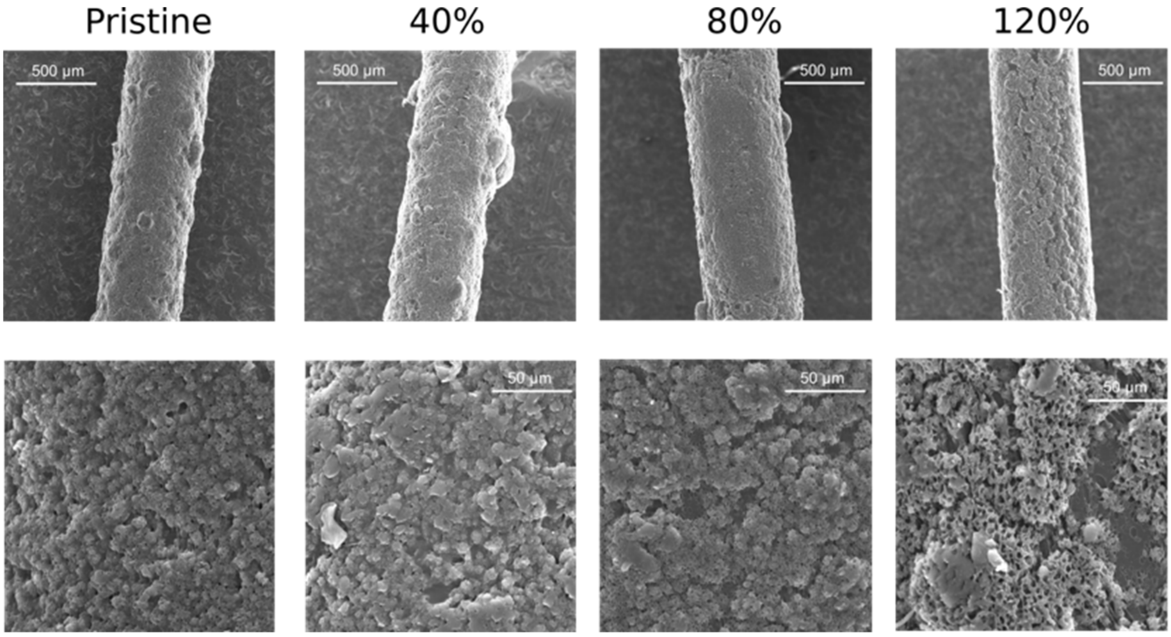

Figure S3. SEM images of 40, 80, and 120% strain profiles for H3078 sensors.

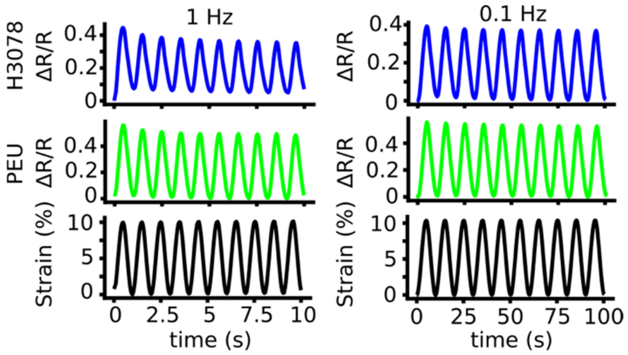

Figure S4. Frequency dependent behaviour of H3078-core sensors strain from 0-10% using a sinusoidal wave pattern A) 1 Hz; B) 0.1 Hz.

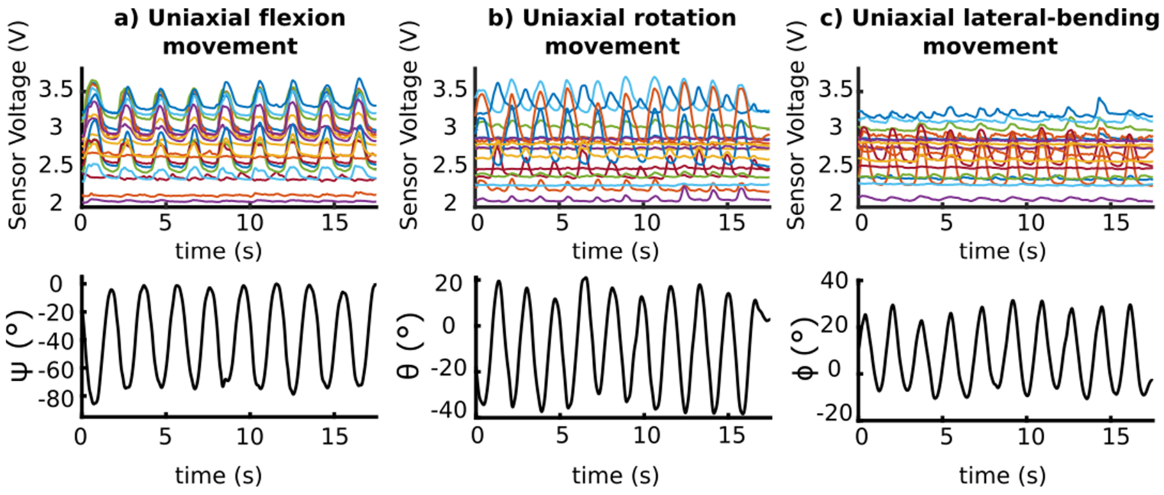

Figure S5. Unnormalized change of strain sensors voltage while performing 3 different types of movement. As a result of the specific sensor placement, voltage change depended on movement type and in each specific movement, only sensors related to that movement were strained. (**a**) Uniaxial flexion movement of the trunk; (**b**) Uniaxial rotation movement of the trunk; (**c**) Uniaxial lateral bending of the trunk.

Table S1. Performance results of the algorithm in the detection of 3 angles of ψ (flexion), θ (rotation), and ϕ (lateral bending) for each participant (P).

	ψ			θ			ϕ		
	R^2	RMSE (deg)	NRMSE (%)	R^2	RMSE (deg)	NRMSE (%)	R^2	RMSE (deg)	NRMSE (%)
**P01**	0.97	2.75	3.21	0.97	2.31	2.87	0.96	2.30	3.35
**P02**	0.92	5.80	5.70	0.91	4.48	4.84	0.90	4.63	6.10
**P03**	0.96	4.07	4.52	0.90	3.84	5.57	0.94	3.97	6.20
**P04**	0.97	2.61	3.89	0.89	2.45	5.90	0.91	2.80	6.47
**P05**	0.95	3.69	5.10	0.90	4.06	5.10	0.88	3.46	6.07
**P06**	0.92	3.71	5.77	0.94	2.78	4.08	0.91	2.71	6.30
**P07**	0.85	4.04	6.64	0.88	3.46	5.54	0.88	3.24	6.30
**P08**	0.91	5.35	6.31	0.92	4.29	4.98	0.88	3.44	6.10
**P09**	0.96	5.37	4.58	0.93	4.32	4.96	0.93	3.63	5.53
**P10**	0.96	4.22	4.24	0.96	3.47	3.66	0.89	3.84	5.30
**P11**	0.94	3.89	5.99	0.96	3.24	3.43	0.93	3.12	5.33
**P12**	0.96	5.59	4.54	0.93	3.57	4.87	0.91	3.70	5.52
**Mean**	0.94 (0.03)	4.26 (1.06)	5.04 (1.05)	0.92 (0.03)	3.52 (0.73)	4.65 (0.94)	0.91 (0.03)	3.40 (0.62)	5.71 (0.85)

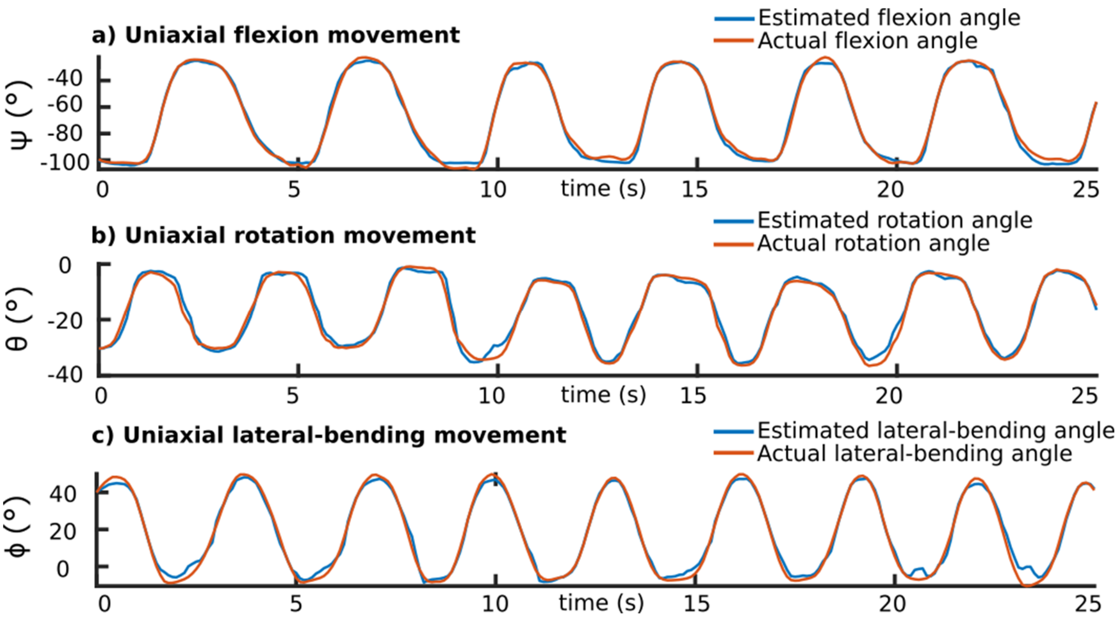

Figure S6. Exemplary comparison between the principal reference and estimated ψ (flexion), θ (rotation), and ϕ (lateral bending) angles in uniaxial movements. (**a**) Uniaxial flexion movement; (**b**) Uniaxial rotation movement; (**c**) Uniaxial lateral bending movement.
